# Purification of *Cyclospora cayetanensis* oocysts obtained from human stool specimens for whole genome sequencing

**DOI:** 10.1186/s13099-018-0272-7

**Published:** 2018-10-12

**Authors:** Yvonne Qvarnstrom, Yuping Wei-Pridgeon, Erik Van Roey, Subin Park, Ganesh Srinivasamoorthy, Fernanda S. Nascimento, Delynn M. Moss, Eldin Talundzic, Michael J. Arrowood

**Affiliations:** 1 Parasitic Disease Branch, Division of Parasitic Diseases and Malaria, Center for Global Health, Centers for Disease Control and Prevention, Atlanta, GA USA; 2 IHRC Inc, Atlanta, GA USA; 3 SRA International, Atlanta, GA USA; 4 Waterborne Disease Prevention Branch, Division of Foodborne, Waterborne, and Environmental Diseases, National Center for Enteric and Zoonotic Infectious Diseases, Centers for Disease Control and Prevention, Atlanta, GA USA; 5 Malaria Branch, Division of Parasitic Diseases and Malaria, Center for Global Health, Centers for Disease Control and Prevention, Atlanta, GA USA

**Keywords:** Whole genome sequencing, Next generation sequencing, *Cyclospora cayetanensis*, Density gradient separation, Flow cytometry sorting, Oocysts

## Abstract

**Background:**

*Cyclospora cayetanensis* is a food-borne intestinal human parasite that causes outbreaks of diarrhea. There is a need for efficient laboratory methods for strain-level characterization to assist in outbreak investigations. By using next generation sequencing, genomic sequences can be obtained and compared to identify potential genotyping markers. However, there is no method available to propagate this parasite in the laboratory. Therefore, genomic DNA must be extracted from oocysts purified from human stool. The objective of this study was to apply optimized methods to purify *C. cayetanensis* oocysts and extract DNA in order to obtain high-quality whole genome sequences with minimum contamination of DNA from other organisms.

**Results:**

Oocysts from 21 human stool specimens were separated from other stool components using discontinuous density gradient centrifugation and purified further by flow cytometry. Genomic DNA was used to construct Ovation Ultralow libraries for Illumina sequencing. MiSeq sequencing reads were taxonomically profiled for contamination, de novo assembled, and mapped to a draft genome available in GenBank to assess the quality of the resulting genomic sequences. Following all purification steps, the majority (81–99%) of sequencing reads were from *C. cayetanensis*. They could be assembled into draft genomes of around 45 MB in length with GC-content of 52%.

**Conclusions:**

Density gradients performed in the presence of a detergent followed by flow cytometry sorting of oocysts yielded sufficient genomic DNA largely free from contamination and suitable for whole genome sequencing of *C. cayetanensis.* The methods described here will facilitate the accumulation of genomic sequences from various samples, which is a prerequisite for the development of typing tools to aid in outbreak investigations.

## Background

*Cyclospora cayetanensis* is a food-borne coccidian pathogen of humans associated with cyclosporiasis outbreaks in the U.S. almost every summer [1–3]. There is little data available on the genetic variation of this parasite. Internal transcribed spacer (ITS) regions between 18S and 28S rRNA genes have been used as molecular typing tools for other organisms. However, ITS1 variability has been reported within individual *C. cayetanensis* oocysts, rendering this region unsuitable for subspecies differentiation [4]. On the other hand, other parts of the *C. cayetanensis* genome, including ribosomal RNA genes, heat shock protein genes, mitochondrial and apicoplast genomes, have little or no sequence variation between samples from various geographical regions [5–8]. A multi-locus sequence typing method based on five microsatellites has been developed [9]. However, this method suffers from low success rate (approximately 50–60%) due to frequent uninterpretable sequence results [9, 10]. More information about *C. cayetanensis* genomic variation between and within various geographical regions are needed to aid investigations of outbreaks and sporadic cases.

There is no method available to propagate *C. cayetanensis* in the laboratory, neither in vitro or in vivo [11]. Therefore, genomic DNA must be extracted from limited human stool specimens collected from clinical cases of cyclosporiasis. Isolating and purifying the transmissible stage of the parasite (oocysts) is complicated by the complexity of stool compositions that vary in each preparation. Moreover, the outer wall of *C. cayetanensis* oocyst is resistant to many commonly used DNA extraction techniques [12]. Next generation sequencing (NGS) has recently been used to obtain draft assemblies of the genome of *C. cayetanensis* from two different geographic regions [13, 14]. These studies were based on genomic sequences obtained from oocysts purified by density gradients and flow cytometry sorting. However, the focus of these publications was on the analysis of the genome sequence data; the descriptions of the laboratory methods to purify the oocysts and obtain genomic DNA were necessarily brief. The present study provides a detailed description of the laboratory methods involved in the genomic sequencing of *C. cayetanensis*. We applied these methods to stool samples from different countries and U.S. outbreaks, collected in three different stool preservatives or transport media, to ensure reproducibility.

## Results

### Discontinuous density gradient purification of oocysts

**Fig. 1:**
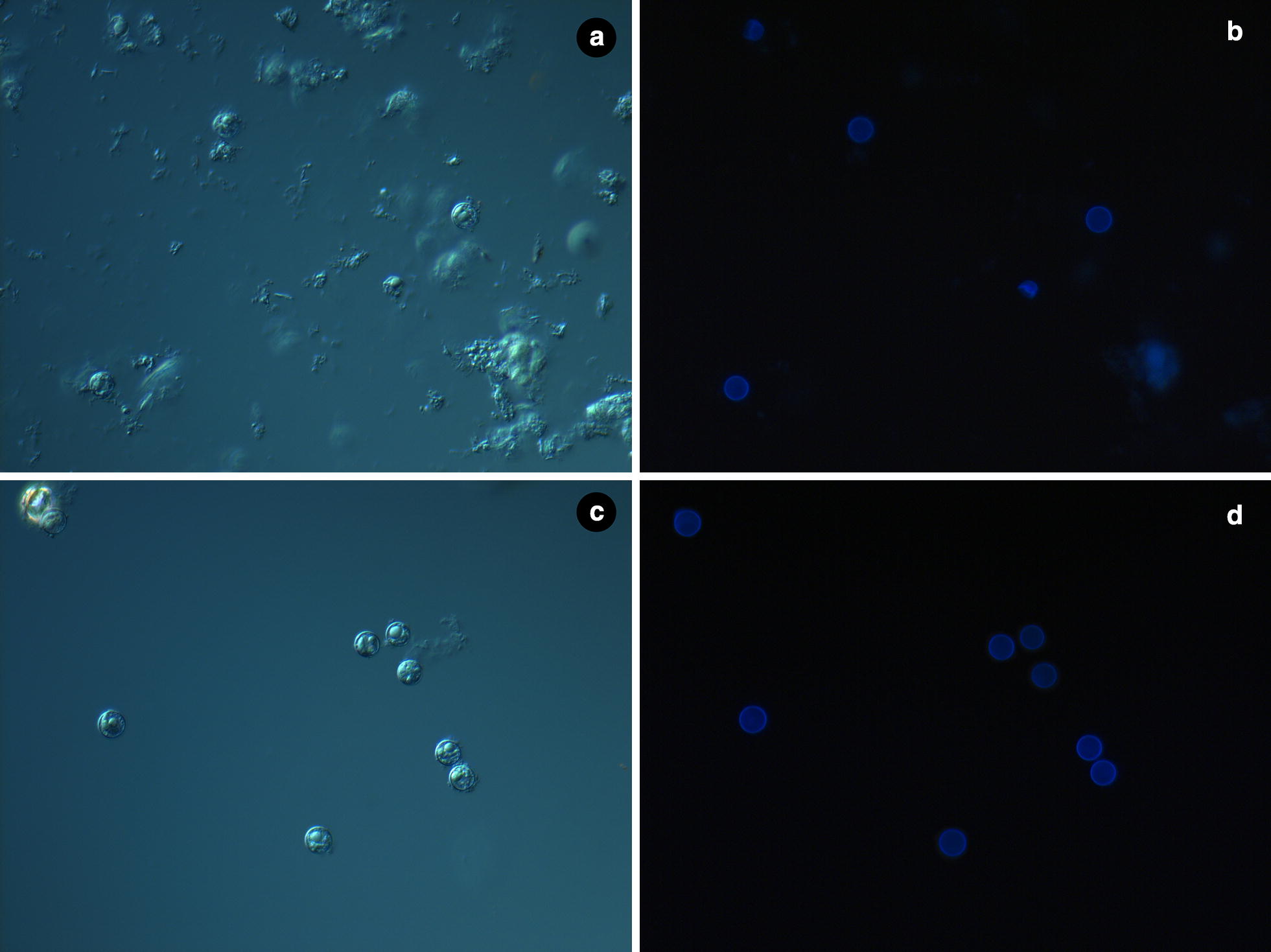
Microscopy images of oocysts after gradient purification without (**a** and **b**) and with (**c** and **d**) Alconox. **a** and **c** Light microscopy; **b** and **d** UV-fluorescence microscopy

The addition of Alconox (final concentration 0.75% w/v) to the gradient purification steps resulted in considerably less contamination (Fig. 1). Lower concentrations of Alconox yielded preparations with more contamination (data not shown). The addition of Alconox benefited the purification of oocysts from stool preserved in potassium dichromate, fixed in a zinc–polyvinyl alcohol (Zn–PVA) based fixative, or collected in a transport medium (Cary-Blair) in our study.

### Separating oocysts from contaminants using flow cytometry

**Fig. 2:**
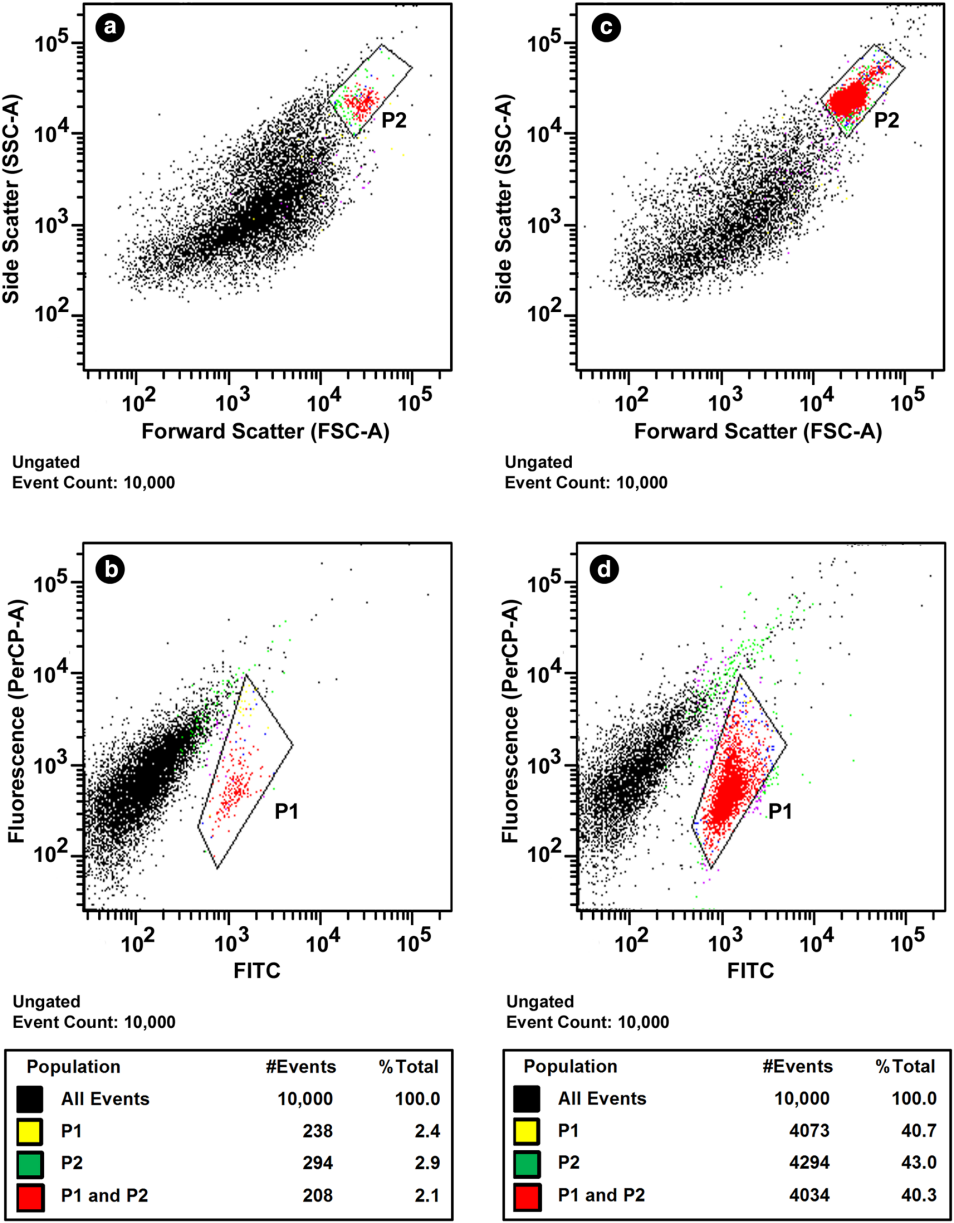
Representative images of flow cytometry sorting of oocysts purified without (**a** and **b**) and with (**c** and **d**) Alconox in the gradient purification process. **a** and **c** Separation based on scatter; **b** and **d** separation based on fluorescence (FITC-A, filter 530/30 nm and PerCP-A, Cy 5.5 filter 695/40 nm). Oocysts (red events in gates P1 and P2) were sorted on logical “AND” function. Both sorted oocysts and sorted oocysts with conflicts were collected in single-cell mode

Gradient purified oocysts were separated from remaining contaminants through flow cytometry sorting. Bi-parameter scatter and fluorescence dot plots of representative oocyst preparations are shown in Fig. 2. Scatter plots, oocyst size and internal complexity alone failed to separate oocysts from all debris (see gate region P2). Therefore, fluorescence dot plots were used to improve the separation (see gate region P1). Flow cytometry sorting was only practical for oocyst preparations that had reached a sufficient degree of purity in the gradient steps; preparations that contained too much stool residue would either clog the instrument or take an exorbitant long time to sort in the flow cytometer. For this reason, samples purified without Alconox were generally less successful in the flow cytometry process: in the example illustrated in Fig. 2, the proportion of oocyst events among all events was only 2.1% for oocysts purified without Alconox (panels a and b), but 40.3% for oocysts purified with Alconox (panels c and d).

### Genomic DNA extraction from purified oocysts

**Fig. 3:**
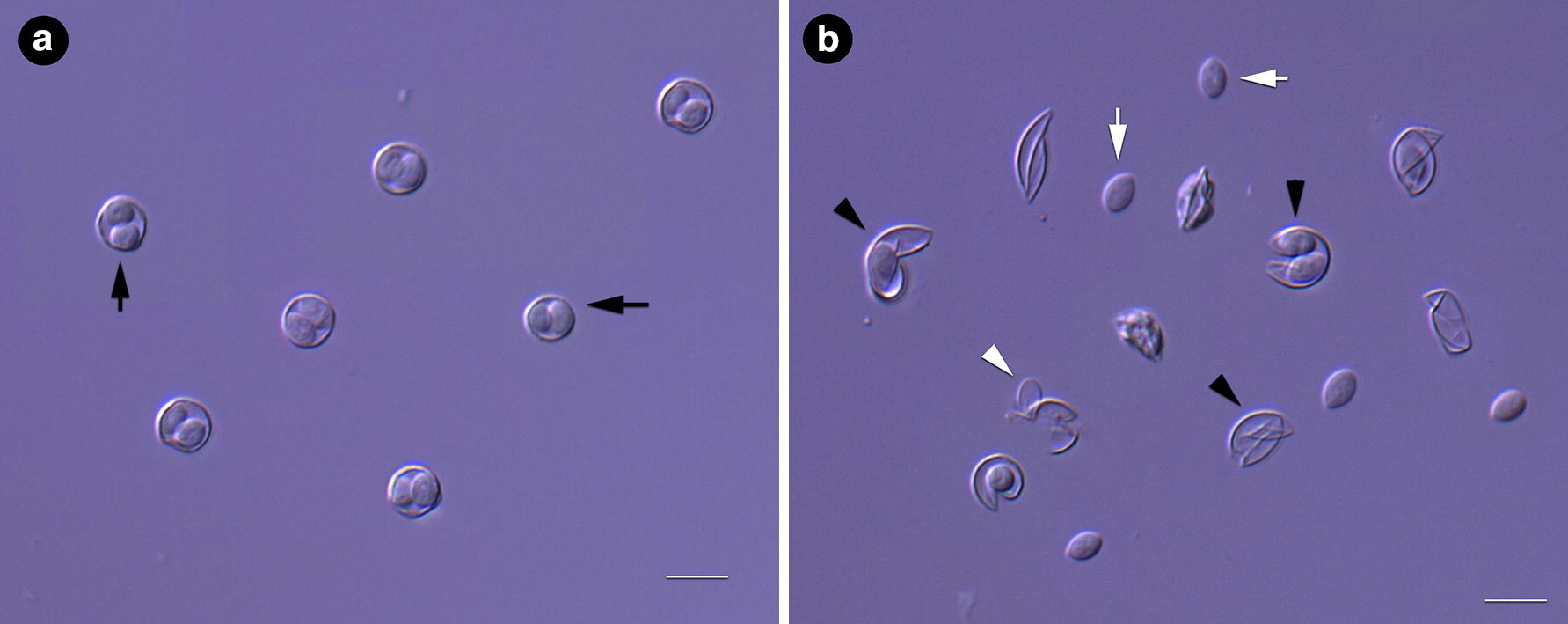
Composite microscopy image of purified oocysts before (**a**) and after (**b**) 15 cycles of freeze and thaw. Black arrows = intact oocysts; black arrowheads = oocysts, partially or completely empty; white arrows = sporocysts; white arrowhead = empty sporocyst. Scale bar = 10 μm

Genomic DNA was extracted from unsorted oocysts as well as sorted oocysts. DNA was extracted using mechanical disruption using freeze-and-thaw cycles. A majority of oocysts needed up to 25 cycles of freeze-and-thaw to disrupt the tough walls of oocysts and sporocysts (where present). Less than 10% of the oocysts showed disruption after 5 cycles of freeze-and-thaw. Purified oocysts before and after 15 cycles of freeze-and-thaw are shown in Fig. 3.

**Fig. 4:**
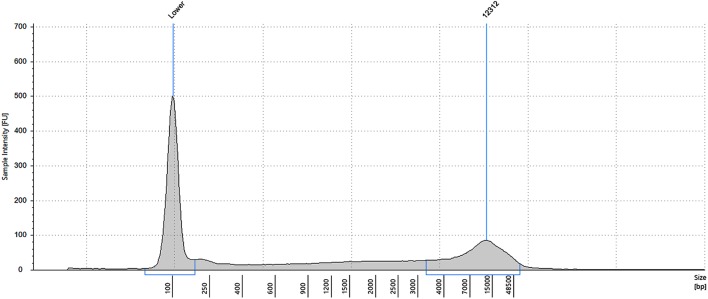
Size distribution of *C. cayetanensis* genomic DNA as measured on a Genomic Screen Tape. DNA was extracted from purified oocysts after 25 cycles of freeze and thaw. The prominent 100 base pair peak is a size marker included in each lane

Yield of genomic DNA extractions ranged from 5 to 15 ng per million oocysts, which was about 10%–30% of the total genomic DNA theoretically present in those oocysts (assuming a haploid genome size of 45 Mb without sporulation). The peak size of genomic DNA extracted using this method was around 12 kb (Fig. 4).

### Bioinformatic analysis of Illumina reads

**Fig. 5:**
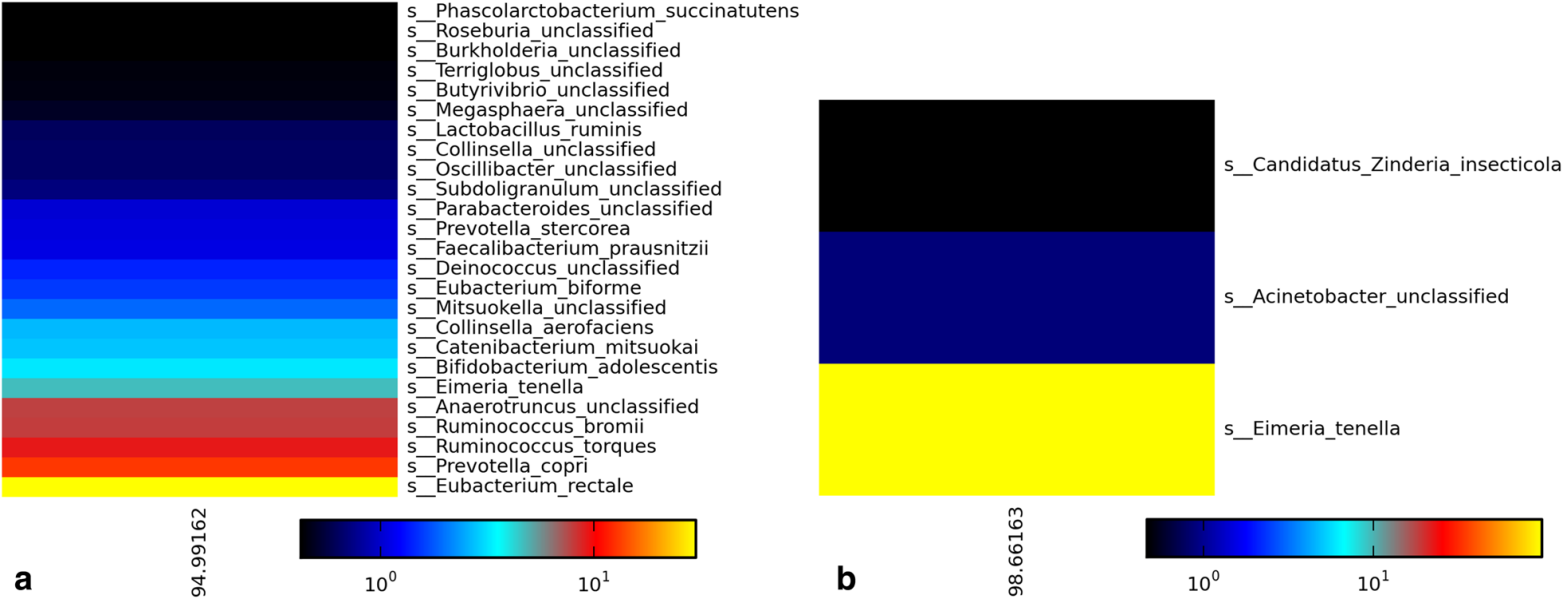
Representative heat maps showing the relative abundance of taxa identified by MetaPhlAn in genomic sequencing datasets obtained from unsorted and sorted oocysts from specimen CDC:HCGM01:97. **a** Before flow-sorting; **b** flow-sorted oocysts. The database used does not contain signatures for *Cyclospora* species. Therefore, reads identified as *Eimeria* and other related eukaryotes could be *C. cayetanensis* sequences

Genomic DNAs from unsorted and sorted oocysts were used to generate Illumina sequencing reads. Trimmed reads were then mapped to the CDC:HCNY16:01 draft genome assembly (Table 1) and analyzed for contaminants by metagenomic profiling (Fig. 5).

**Table 1:** Proportion of trimmed reads mapped to the CDC:HCNY16:01 draft genome assembly using oocysts from three representative samples

Purification status of oocysts	Number of mapped reads/total number of reads = % of reads mapped to HCNY assembly		
	CDC:HCRI01:97	CDC:HCGM01:97	CDC:HCTX69:14
Before flow sorting	2,262,396/9,739,022 = 23%	1,781,583/9,315,212 = 19%	163,179/2,767,726 = 6%
Flow cytometry sorted	6,282,480/7,785,610 = 81%	5,954,094/6,137,902 = 97%	2,486,477/2,888,1657 = 86%

Flow cytometry sorting had a major impact on the quality of the resulting Illumina sequencing data. Metagenomic profiling analysis revealed that the majority of reads from unsorted oocysts matched prokaryotic sequences, whereas relatively few prokaryotic sequences were identified in the flow-sorted oocysts (Fig. 5a and b, respectively). Furthermore, less than 25% of the reads from un-sorted oocysts could be mapped to the CDC:HCNY16:01 draft genome assembly, whereas more than 80% of the reads from sorted oocysts could be mapped to the same genome draft (Table 1).

### De novo assembly of draft genomes of *C. cayetanensis* samples

DNA extracted from flow-sorted oocysts were used to obtain draft genome assemblies. Reads from Illumina libraries with different insert sizes were pooled together and assembled de novo. A comparison of these new genome assemblies is summarized in Table 2. The new assemblies had contig numbers between 669 and 2827. The total length of these contigs and the GC content were consistent with published draft genome assemblies of *C. cayetanensis* strains CDC:HCNY16:01 and CHN_HEN01 (GenBank accession numbers ASM130573v1 and ASM76915v2).

**Table 2:** Summary statistics of *de novo* assemblies (including scaffolded regions) obtained from *C. cayetanensis* samples, including the two previously published assemblies (two first rows)

	Total sequence length (bp)	Number of contigs (> 500 bp)	Contig N50	Contig L50	G+C content (%)
CHN_HEN01 [14]	44,034,411	3573	43,794	282	52
HCNY16:01 [13]	44,563,857	865	187,023	72	52
HCDC004_96	44,485,136	1120	142,454	92	52
HCGM002_97	44,562,363	1073	145,614	91	52
HCGM011_97	44,518,937	677	235,157	64	52
HCGM012_97	44,435,629	2796	37,553	352	52
HCNP016_97	44,777,959	2659	62,977	203	52
HCRI001_97	45,472,771	1526	136,687	101	51
HCTX119_13	43,837,200	1503	83,084	160	52
HCTX365_13	44,340,945	1646	86,734	155	52
HCJK001_14	44,286,668	669	180,727	72	52
HCTX535_14	44,523,203	1266	130,032	104	52
HCTX569_14	44,347,388	792	202,065	71	52
HCJK008_15	44,551,431	1570	104,384	140	52
HCJK011_15	44,458,373	1289	112,390	114	52
HCJK015_15	44,350,146	2827	36,648	354	52
HCTX542_15	44,626,029	1426	147,450	94	52
HCTX547_15	44,368,440	1711	81,750	169	52
HCMX010_16	44,405,937	816	218,769	59	52
HCNE181_16	44,372,150	1421	100,417	134	52
HCTX460_16	44,233,679	1640	82,105	162	52
HCTX495_16	44,243,943	2776	38,624	336	52
HCTX503_16	44,362,393	1790	79,041	181	52

## Discussion

Next generation sequencing (NGS) was recently used to obtain draft genome assemblies of *C. cayetanensis*, providing opportunities to explore metabolism, pathogenicity, and genetic variation of this parasite [9, 10, 13, 14]. These studies were performed using similar methods as presented in this study, but the publications only briefly mention the laboratory methods involved without enough specifics to allow other researchers to replicate them. Here we described the detailed laboratory methods for purifying and processing oocysts from stool to obtain high quality NGS data.

A previous study has concluded that a detachment solution (containing disodium pyrophosphate) can improve the recovery of *C. cayetanensis* from stool during density gradient purification compared to 0.01% Tween 20 [15]. Moreover, the addition of 0.1% of detergent Alconox in the wash solution can improve oocyst recovery from environmental samples [16]. In this study, we found that the addition of 0.75% of Alconox could substantially improve the separation of oocysts from fecal contaminants during gradient purifications. The use of Alconox resulted in purification that was more efficient and therefore applicable to a wider range of stools, including those with low oocyst counts.

Partially purified oocysts can be further separated from contaminants using flow cytometry sorting. Flow cytometry was successfully used to separate *Cyclospora* oocysts from stool debris in previous studies [17, 18]. In this study, three factors facilitated the efficient separation of oocysts from stool debris using flow cytometry: First, oocysts exhibit autofluorescence. Second, PI staining helped discriminate debris from oocysts since the latter were not PI stained. Third, Alconox used in the density gradient purification greatly reduced contaminant load prior to flow cytometry sorting; specimens purified without Alconox contained more debris that slowed down or inhibited the flow cytometry process. A 488 nm laser with fluorescence filters appropriate for FITC and PerCp-Cy5.5 were used to separate oocysts from debris for all four specimens included in this study. However, depending on the particular composition of contaminants, sorting of other specimens may benefit from the use of alternative filter sets appropriate for PI. We have successfully used filters for r-phycoerythrin (PE), PE-Texas Red, and PE-Cy7 in the past. Lasers with shorter wavelengths (violet and UV) can enhance separation since oocyst autofluorescence is shifted even further from the debris (data not shown).

Draft genome assemblies obtained from purified oocysts in this study had comparable assembly statistics to the two previously published assemblies of *C. cayetanensis*, samples CDC:HCNY16:01 and the CHN_HEN01 [13, 14]. Sample CDC:HCNY16:01 was processed using the same methods as described in this study. The assembly for CHN_HEN01 was obtained using other methods for library preparation and sequencing (454 GS-FLX complemented with Illumina 100 cycles) but similar methods for purifying and extracting DNA from oocysts (excluding Alconox treatment). These findings indicate that the laboratory methods described here are reproducible and generally result in good quality genome assemblies of *C. cayetanensis*.

## Conclusions

Laboratory methods were applied to obtain *C. cayetanensis* genomic sequences using human stool specimens as starting material. A key step to obtaining good quality genomic sequences was flow cytometry sorting of the oocysts to remove contaminants. The addition of Alconox in the discontinuous gradient purification steps greatly improved the purification efficiency and thereby enabled the flow cytometry process for a wider range of stools. The genomic drafts obtained in this study represented at least seven separate U.S. outbreaks and four different countries, providing a good starting material for exploring the genetic diversity of this parasite.

## Methods

### Stool specimens

Twenty-one human stool specimens were selected for this study due to the presence of relatively high numbers of oocysts (large volume and/or high parasite load as estimated by UV-fluorescence microscopy). We included twelve specimens collected from seven separate U.S. outbreaks: Washington D.C. in 1996 (HCDC004_96); Rhode Island in 1997 (HCRI001_97); Nebraska in 2016 (HCNE181_16); and Texas in 2013 (HCTX119_13 and HCTX365_13), 2014 (HCTX535_14 and HCTX569_14), 2015 (HCTX542_15 and HCTX547_15) and 2016 (HCTX460_16, HCTX495_16 and HCTX503_16). Also included were 9 specimens collected in four different countries: Guatemala (HCGM002_97, HCGM011_97 and (HCGM012_97), Nepal (HCNP016_97), Indonesia (HCJK001_14, HCJK008_15, HCJK011_15 and HCJK015_15), and Mexico (HCMX010_16). Specimens collected prior to 2013 and specimens collected in countries outside the U.S. were preserved in 2.5% (w/v) aqueous potassium dichromate and stored at 4 °C following collection (n = 11). Specimen HCNE181_16 was collected in Cary-Blair transport medium. The remaining specimens (n = 9) were collected in Zn–PVA.

#### Discontinuous density gradient purification of oocysts from stool

Preservatives were removed by centrifugation (3200×*g*) at 4 °C for 10 min and decanting the supernatant. The resulting pellets were washed with 0.01 M phosphate buffered saline (PBS, pH 7.2). Stool suspensions were passed through a disposable 125 µm flat sieve (e.g. SATA RPS^®^ 0.3 L filter 1010420, SATA USA, Spring Valley, MN) to remove large particles, centrifuged as described above and re-suspended in PBS at a 1:3 ratio (v/v). Samples were gently mixed (to avoid foaming) with an equal volume of 1.5% (w/v) Alconox detergent solution (Alconox Inc., White Plains, NY) and subjected to discontinuous sucrose gradient purification as previously described for *Cryptosporidium* [19, 20] with the following modifications for *Cyclospora* purification. In the sucrose gradient centrifugation step, *Cyclospora* oocysts accumulated at the interface between the two sucrose layers (the high-density fraction), as well as the interface between the sample overlay and the top sucrose layer (the low-density fraction). Each of these fractions were collected separately, diluted to three times its volume with deionized water (dH_2_O), and centrifuged (3200×*g* for 10 min) to pellet oocysts. The pellets were then re-suspended in PBS to half of the original volume and then gently mixed with 1.5% Alconox to a final concentration of 0.75%. The sucrose gradient purification was repeated once more. The sucrose gradient-purified oocyst pellets were diluted with PBS at a ratio of approximately 1:6 (v/v).

Sucrose gradient purified oocysts were then subjected to cesium chloride gradient purification as previously described [19] with one critical modification. The oocyst-containing fraction of the gradient (~ 1 ml collected from the interface between the sample layer and the cesium chloride layer) was diluted with dH_2_O to approximately three times the starting volume (i.e., to ~ 3 ml total volume or more) and centrifuged (16,300×*g* for 3 min) to pellet oocysts. Pelleted oocysts were re-suspended in PBS, pooled together, and quantified using an improved Neubauer hemacytometer (Hausser Scientific, Horsham, PA).

### Flow cytometry sorting

Gradient-purified *C. cayetanensis* oocysts were sorted by flow cytometry using a BD FACSAria III (BD Biosciences, San Jose, CA) equipped with blue (488 nm) and red (633 nm) lasers. Oocysts were diluted with sheath fluid appropriately for a sorting efficiency rate ≥ 45% and processed in the single-cell sorting mode using a 70 µm nozzle at 70 psi. Propidium iodide (PI) was added to the oocyst preparation at a final concentration of 1.0 µg/ml to label “dead” cells by binding to their DNA, thus increasing the shift away from oocysts because oocysts do not take up PI due to their intact oocyst wall [21]. Oocysts were identified by their size (approximately 8–10 µm) by forward scatter (FSC), their internal complexity by side scatter (SSC), and their autofluorescence emission properties when excited by 488 nm light using fluorescence filters appropriate for fluorescein isothiocyanate (FITC), tandem fluorochrome peridinin chlorophyll protein, and cyanine 5.5 (PerCP-Cy5.5).

### Genomic DNA extraction

Twenty-five cycles of freeze-and-thaw (freeze for 1 min by immersion in an ethanol/dry ice bath followed by thawing at 95 °C for 1 min in a heat block) was used to mechanically break open the purified oocysts. Genomic DNA was extracted from ruptured oocysts using DNeasy Blood & Tissue Kit (Qiagen, Germantown, MD) following the manufacturer’s instructions. The concentration of extracted genomic DNA was determined using a Qubit dsDNA HS Assay Kit (Thermo Fisher Scientific Inc., Cleveland, OH). The integrity of genomic DNA was analyzed by electrophoresis using Genomic DNA Analysis ScreenTape on a 2200 TapeStation (Agilent Technologies, Santa Clara, CA).

### Genomic DNA library construction and Illumina sequencing

Genomic DNA (10 ng) was sheared in an M220 Focused-ultrasonicator™ (Covaris Inc., Woburn, MA) using settings for an average fragment of 300, 500, or 700 base pairs. Genomic libraries were constructed for Illumina sequencing using Ovation^®^ Ultralow Library Systems V2 (NuGEN Technologies Inc., San Carlos, CA) [22]. Libraries were barcoded to facilitate pooling during subsequent sequencing runs. Size distribution and concentration of genomic DNA libraries were analyzed by electrophoresis using Genomic DNA Analysis ScreenTape and D1000 ScreenTape on a 2200 TapeStation (Agilent). Barcoded genomic DNA libraries were pair-end sequenced using Illumina MiSeq Reagent v3 (600 cycles, 2 × 300 bp) kits (Illumina Inc., San Diego, CA).

### Bioinformatic analysis

Raw sequence data were assessed for quality using FASTQC v0.11.5. AdaptorRemoval v2.2.2 [23] was used to remove adaptor sequences from reads and to merge overlapping paired reads into consensus sequences. Genome assembly was performed using SPAdes v3.12.0 [24]. Contigs derived from prokaryotic human gut microbiota were removed with BBMap v35.82 [25]. CLC Genomics Workbench Map to Reference assembler (QIAGEN) was used to map reads to the draft genome assembly of strain CDC:HCNY16:01 (GenBank Accession No. ASM130573v1).

Metagenomic analysis was performed to estimate the degree of contamination in the sequences obtained at various steps of oocyst purification. Sequencing reads were profiled for taxonomic diversity by assessing the percentage of data arising from certain contaminating organisms using MetaPhlAn [26] and signature database version 2.0. This database does not contain signatures from *Cyclospora* species.
